# Applying the resource dispersion hypothesis to a fission–fusion society: A case study of the African lion (*Panthera leo*)

**DOI:** 10.1002/ece3.5456

**Published:** 2019-07-17

**Authors:** Moreangels M. Mbizah, Marion Valeix, David W. Macdonald, Andrew J. Loveridge

**Affiliations:** ^1^ Wildlife Conservation Research Unit (WildCRU), Department of Zoology, Recanati‐Kaplan Centre University of Oxford Oxford UK; ^2^ CNRS, Laboratoire de Biométrie et Biologie Evolutive, CNRS UMR 5558 Université Claude Bernard Lyon 1 Villeurbanne France; ^3^ CNRS HERD (Hwange Environmental Research Development) Program LTSER France, Zone Atelier “Hwange” Dete Zimbabwe

**Keywords:** fission–fusion, group size, heterogeneity, lion, patch richness, prey availability, resource dispersion hypothesis, sociality

## Abstract

The relationship between the spatiotemporal distribution of resources and patterns of sociality is widely discussed. While the resource dispersion hypothesis (RDH) was formulated to explain why animals sometimes live in groups from which they derive no obvious benefits, it has also been successfully applied to species that benefit from group living. Some empirical tests have supported the RDH, but others have not, so conclusions remain equivocal and further research is required to determine the extent to which RDH predictions hold in natural systems. Here, we test four predictions of the RDH in an African lion population in the context of their fission–fusion society. We analyzed data on group composition of GPS‐collared lions and patterns of prey availability. Our results supported the first and second predictions of the RDH: Home range size (a) was independent of group size and (b) increased with distance between encounters with prey herds. Nonetheless, the third and fourth RDH predictions were not supported: (c) The measure of resource heterogeneity and (d) resource patch richness measured through prey herd size and body size had no significant effect on lion group size. However, regarding the fourth prediction, we added an adaptation to account for dynamics of fission–fusion society and found that the frequency of pride fission increased as group size increased. Our data set restricted us from going on to explore the effect of fission–fusion dynamics on the relationship between group size and patch richness. However, this should be investigated in future studies as including fission–fusion dynamics provides a more nuanced, realistic appreciation of lion society. Our study emphasizes the importance of understanding the complexity of a species' behavioral ecology within the framework of resource dispersion. Whatever larger theoretical framework may emerge to explain lion society, incorporating fission–fusion dynamics should allow the RDH to be refined and improved.

## 1 INTRODUCTION

The selective advantage of group living is, in many social animals, attributed to the direct benefits of cooperation, such as cooperative foraging, alloparental care, or territorial defense (Krause & Ruxton, 2002). However, some animals live in groups but travel and hunt alone and show less obvious benefits of group living, as demonstrated in some populations of European badgers (*Meles meles*; Kruuk, 1978), red foxes (*Vulpes vulpe*s; Macdonald, 1981), brown hyenas (*Hyaena brunnea*; Mills, 1982), and giant otters (*Pteronura brasiliensis*; Groenendijk et al., 2015). The resource dispersion hypothesis (RDH) describes how groups may form even in the absence of any functional advantage to any individual from the presence of another. It links ecological factors, such as spatiotemporal patterns in the richness and dispersion with which resources become available, with sociological characteristics, such as the size of social groups and the extent of their territories (Carr & Macdonald, 1986; Macdonald, 1983). The RDH predicts that where resources are heterogeneously distributed in space and time, individuals have to defend a large enough territory to guarantee that there are always sufficient resource patches available and this allows several individuals to share the same resources without imposing intolerable, if any, costs on each other (Carr & Macdonald, 1986; Macdonald, 1983). The RDH offers four general predictions. The first prediction is that territory size (home range size in this study) is independent of group size. This prediction is a consequence of the subsequent predictions as different aspects of resource availability are expected to affect territory size and group size independently. On the one hand, the second prediction of the RDH is that the dispersion of resources determines territory size. The basic concept is that even a single animal using patchy resources will have to defend a large enough area to be sure that at least one patch will be available to satisfy its resource requirements. On the other hand, resource richness and heterogeneity have been described as part of the environmental parameters that determine the probability with which additional group members can be sustained in a territory (Carr & Macdonald, 1986). This leads to the RDH third and fourth predictions: Group size is determined by the heterogeneity of available resources, and group size is determined by the richness of available resources (Carr & Macdonald, 1986; Macdonald, 1983; Macdonald & Johnson, 2015). All else being equal, if patch richness and resource heterogeneity increase, then the costs to primary occupants of tolerating additional group members in their territory diminish (Macdonald & Johnson, 2015).

While the RDH is recognized as a potential explanation for grouping behavior in animals, there has been particular debate around the prediction that patch richness is positively related to group size (see Macdonald & Johnson, 2015 for a review). Here, we argue that the fission–fusion dynamics of some social species may shed light on this debate. Fission–fusion dynamics, whereby group membership is not spatiotemporally stable, have been shown to occur in the majority of group living animals (Silk, Croft, Tregenza, & Bearhop, 2014). The size and composition of these groups change frequently as groups split (fission) or merge (fusion) (Couzin & Laidre, 2009). This allows individuals to adjust in particular to environmental and social conditions (Holmes, Gordon, Louis, & Johnson, 2016). For instance, the development of fission–fusion social organization in both the spider monkey (*Ateles paniscus chamek*) and chimpanzee (*Pan troglodytes*) has been linked to the high level of feeding competition between females within a group, caused by the spatial and temporal patchiness in food dispersion and abundance (Symington, 1990). Sueur et al. (2011) attribute fission–fusion dynamics in avian systems to variation in the environment across seasonal diel and tidal cycles. Fission–fusion has also been described as an adaptive outcome where individuals in a group fail to reach a consensus, that is, group members fail to follow the same action (Conradt & Roper, 2005; Kerth, Ebert, & Schmidtke, 2006). Given that patterns of sociality and resource distribution have an effect on fission–fusion dynamics, it is therefore important to understand how the fission–fusion dynamics can subsequently influence the relationship between prey availability and sociality (Silk et al., 2014).

Here, we test the four predictions of the RDH and also take into account the relationship between fission–fusion dynamics and sociality. We based these tests on field data collected on African lions (*Panthera leo*) in Hwange National Park, Zimbabwe. Lions live in social groups of between two and eighteen related females, their dependent offspring, and a coalition of adult males (Packer & Pusey, 1982). Although lions are widely said to be the most conspicuously social of felids, pride members are not always together and a process of fission–fusion commonly results in the pride splitting into smaller subgroups (Packer & Pusey, 1997; Schaller, 1972). Schaller (1972) speculated that the spatial and temporal variability of resources in heterogeneous landscapes caused these fission–fusion dynamics. Water sources might be a critical resource for lions not because lions drink there, but because their prey aggregate there to access water (Redfern, Grant, Biggs, & Getz, 2003; Valeix et al., 2009). Indeed, river confluences in the Serengeti National Park, Tanzania, and waterholes in Hwange National Park, Zimbabwe, have emerged as important resource patches for lions (see Mosser, Fryxell, Eberly, & Packer, 2009; Valeix, Loveridge, & Macdonald, 2012, respectively). These studies found resource patch heterogeneity to be a prerequisite to the development of group territoriality and that in poor quality habitats; animals exclude each other from richer patches. Our study now seeks to build on this growing body of knowledge but differs from its predecessors by taking into account for the first time the role of fission–fusion dynamics, which is characteristic of lion sociality. We also measured prey distribution directly and at the home range scale instead of using water sources as proxies, since lions also make kills in other parts of their home ranges far from water sources. Lastly, we broke down prey availability into three measures (abundance, dispersion, and richness) to better understand the effects of prey availability. Further, patch richness in our system was approximated by two measures: prey herd size and prey body size (see below for further details).

Field tests of RDH are few because the data necessary are not readily gathered (Johnson, Kays, Blackwell, & Macdonald, 2002). However, the natural spatial heterogeneity in prey abundance and dispersion in Hwange National Park provided us with a good opportunity to test the four main predictions from the RDH (Table 1). Knowing that lions live in prides that can exhibit fission–fusion dynamics, we expected RDH principles to lead to relationships between measures of patch richness and group size, but we saw no apparent reason to predict whether that relationship would manifest with respect to total pride size or to the size of fissioned hunting parties (subgroups). Therefore, following the fourth prediction, which used the simplest, least nuanced metric of total group size, we next sought to deconstruct this crude measure into a more naturalistically realistic exploration of the relationship between the richness of resource patches and group size by taking into account how fission–fusion dynamics may affect this relationship. However, because of the small sample size, we failed to test this interaction and could only examine the relationship between frequency of pride fission and group size (Table 1).

**Table 1:** Summary of the RDH predictions and the results of this study

Predictions	Results	Comments
(i) Home range size is independent of group size.	Both pride size and mean subgroup size had no effect on home range size.	First prediction of the RDH is supported.
(ii) Home range size increases with increase in resource dispersion.	Home range size increased as the mean distance between encounters with prey herds increased.	Second prediction of the RDH is supported.
(iii) Group size increases as resource heterogeneity increases.	The index of resource heterogeneity had no effect on pride size and mean subgroup size.	Third prediction of the RDH is not supported.
(iv) Group size increases as the richness of resource patches increases.	Both indices of patch richness had no influence on pride size and mean subgroup size.	Fourth prediction of the RDH is not supported.
(v) Fission–fusion dynamics is influenced by group size.	The frequency of pride fission increased with increase in pride size.	Fission–fusion dynamics is an adaptive mechanism for dealing with social and environmental conditions, which will need to be considered in future studies of RDH.

## 2 MATERIALS AND METHODS

### 2.1 Study area

**Figure 1:**
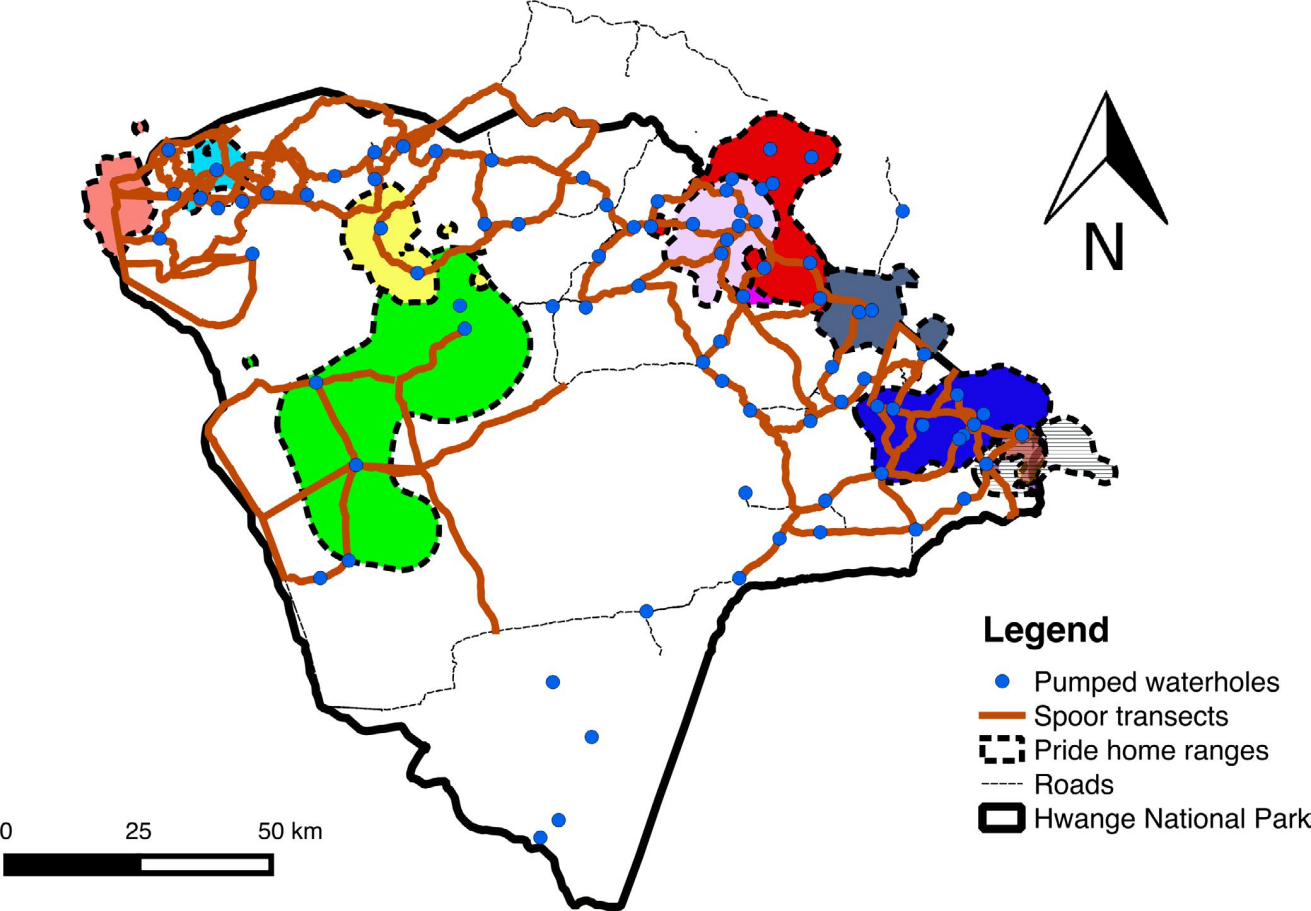
Map of Hwange National Park showing the location of pumped waterholes, spoor transects, and lion pride home ranges

Hwange National Park is a semiarid dystrophic savannah on Kalahari sands, on the northwestern border of Zimbabwe, and it covers approximately 15,000 km^2^. The east and southern parts of the park are dominated by open‐wooded savannas on Kalahari sands, primarily teak woodland (*Baikiaea plurijuga)* and *Combretum/Terminalia* woodlands. Batoka basalt and Karoo sediments in the north and northwest of the park are dominated by *Colophospermum mopane* woodlands interspersed with grassland vleis. Most rain falls between November and February, when water is largely available to animals in waterholes, rivers, and pools and is very unlikely to constrain space use of water‐dependent herbivores. Natural surface water then becomes scarce as the dry season progresses and only pumped waterholes (~50), mostly in the north of the park to maintain water availability (Figure 1). Besides, at the end of dry season, both browsing and grazing resources are of the lowest quality. These differences in vegetation and water distribution across the park result in differences in the distribution of herbivores in terms of both assemblages and abundance (Chamaillé‐Jammes, Charbonnel, Dray, Madzikanda, & Fritz, 2016). We therefore commonly distinguish three seasons in Hwange National Park: the wet season (November–February), the early dry season (March–June), and the late dry season (July–October).

### 2.2 Lion data

We used data from 12 female lions, all from different prides across the park, equipped with GPS radio‐collars between 2013 and 2015. The lions’ locations were available every two hours, day and night. Positional data from the GPS radio‐collars were downloaded regularly, and for this study, we used lion location data for the early dry season and late dry season (which corresponds to periods when prey data were available). GPS radio‐collared lions were regularly tracked to record pride composition, but at times the sightings were opportunistic. Given the difficulty to see lions in the wooded savanna of Hwange and of the large study area to be covered, we managed to see each pride on average 13.8 times per season (range: 7–49 times per season), over three years. For each lion sighting, we recorded the number of individuals present, and the identity, age, and sex of the individuals. We looked at two aspects of lion group size: (a) pride size (total number of adults in a pride obtained from a long‐term lion sightings database—since 1997—with accurate records of the individuals within the study lion prides) and (b) mean subgroup size (mean number of adults observed at each sighting). For each pride, we also noted the reproductive state of the females; a female with cubs was recorded as breeding and a female without cubs as not breeding. Each lion sighting was recorded as a fission–fusion event and all the lion sighting data were used to calculate the number of times the pride was seen in subgroups as a percentage of the total number of pride and subgroup sightings, which we considered as a proxy of the frequency of pride fission. Home ranges were defined as the 90% probability contour of location distribution using the fixed kernel density estimator (Powell, 2000) and the reference smoothing factor href (Hemson et al., 2005; Figure 1). Only seasons for which GPS receivers were operational during the whole season were used to calculate seasonal home ranges (4‐month periods). The mean number of relocations for collared lions was 1,317 per season (range: 681–1,475). Home range analysis was done using the AdehabitatHR package (Calenge, 2006) in the statistical software R (R Core Team, 2019).

### 2.3 Prey data

Prey availability was assessed using the spoor counting method, which involves counting the tracks made by animals when they cross the roads. This is an indirect method of estimating population abundance and assumes that the intensity or frequency of animal tracks is correlated to population size (Wilson & Delahay, 2001). Spoor counting has been extensively used for estimating carnivore abundance (Funston et al., 2010) and has been found to be reliable for estimating abundance of large herbivores as well (Silveira, Jacomo, & Diniz, 2003). Multispecies spoor count surveys were conducted from 2013 to 2015 during the early dry season and the late dry season. Most of the available roads in the study area were used as transects (*n* = 64 transects) and were between 9 and 55 km long (Figure 1). The 64 selected transects were within areas that lions frequent. These transects were driven and spoor identified with the help of highly skilled and experienced trackers, and care was taken to avoid double counting spoor. Vehicles driven at a speed of 10–15 km/hr served as an observation platform during spoor surveys, with a driver, a recorder, and a tracker sitting on a customized seat mounted on the front of a vehicle. Roads were not swept to remove old spoors before our spoor counting. We could only carry out the surveys once per season because of logistical constraints, as the monitored area is huge (7,109 km^2^); we opted to construct surveys over a wide range of prey availabilities, thereby limiting the opportunity for replicates.

When fresh spoor (<24 hr old) was encountered, it was assessed for species, herd size, age class, and sex. The experienced trackers were able to determine whether the spoor was fresh by the state and detail of the spoor. The shape and size of the spoor aided in determining the species, its age, and sex. The number of spoors around that area was counted to estimate the herd size, and these were counted separately for each species in cases of mixed‐species herds. Only herbivore species and herd size were used in the analyses, and we are confident that our highly skilled and experienced trackers could reliably assess these. Spoor were counted if they crossed transects, but subsequent recrossings were ignored when the trackers judged from the animal's movement patterns that these were apparently made by the same animal. During the surveys, spoor of a range of herbivores and carnivores were identified to the species level, but only spoor from lion prey species were used in this study. Prey species included in the analysis were Burchell's zebra (*Equus quagga*), giraffe (*Giraffa camelopardalis*), greater kudu (*Tragelaphus strepsiceros*), impala (*Aepyceros melampus*), warthog (*Phacochoerus aethiopicus*), steenbok (*Raphicerus campestris*), common duiker (*Sylvicapra grimmia*), sable (*Hippotragus niger*), roan antelope (*Hippotragus equinus*), buffalo (*Syncerus caffer*), eland (*Taurotragus oryx*), and juvenile African elephant (*Loxodonta Africana*) (frequently recorded as prey during drought years in Hwange; Loveridge, Hunt, Murindagomo, & Macdonald, 2006).

The spoor survey data were overlaid on lion home ranges in QGIS (QGIS Development Team, 2019), and the spoor transects that fell within each lion home range were clipped. Lion prey species examined in this study are gregarious, and each prey herd represents a patch available to lions and the different characteristics of the herd such as prey body size and herd size characterize the richness of the patch. For each home range and each season, we assessed four measures of prey availability (all prey species were pooled for the analyses): one index of prey dispersion (number of km/prey herd, which describes the distance lions have to travel to encounter prey or the effort involved in searching for prey [Valeix et al., 2010]), one index of resource heterogeneity, which describes the variability in which lions encounter patches of different levels of richness (coefficient of variation in the distance between prey herds), and two indices of patch richness. We considered both mean prey herd size and mean prey body size as potential proxies of patch richness. Indeed, a larger herd might be considered a richer patch insofar as, all else being equal, it provides an opportunity for more than one lion to make a kill (Schaller, 1972) and a higher chance of the lions finding a vulnerable individual within the herd. Additionally, the mean prey body size represents the quality of the food resource in terms of the energy gained from consumption of that prey species (Carr & Macdonald, 1986). Large‐bodied prey might be considered a rich patch insofar as, for example, a single eland or giraffe could readily feed several lions, whereas a single gazelle could not (see Schaller, 1972; Macdonald & Johnson, 2015). Prey body size was calculated using the average adult female body mass obtained from Cumming and Cumming (2003).

### 2.4 Statistical analysis

Statistical analyses were run using the package MASS (Venables & Ripley, 2002) in R version 3.5.2 (R Core Team, 2019). Large herbivores are often seen in large herds and this might suggest that prey body size and herd size are correlated. We therefore did a preliminary analysis to assess the correlation (Pearson's correlation) between mean prey body size and mean prey herd size. We found no significant correlation between these two variables (*r* = .40; *n* = 12; *p* = .20), suggesting that large prey do not necessarily occur in large herds or small prey in small herds; therefore, we included both variables in the analyses. We used logistic regression to preliminarily examine the role of reproductive state on grouping patterns as pride females tend to band together to share the responsibilities of nursing and protecting the pride's young (Packer & Pusey, 1997). We nonetheless found no significant relationship between pride size and reproductive state (estimate ± *SE* = .074 ± .123; *t*(10) = 0.60; *p* = .56). These results may be because nearly all prides (83%) had cubs. Data were first tested for normality using the Shapiro–Wilk test, and home range size was subjected to a logarithmic transformation to reach normality requirements. The small sample sizes restricted our analyses to simple/univariate analyses. To test the first RDH prediction, we fitted two linear models: home range size~pride size and home range size~mean subgroup size. To test the second RDH prediction, we fitted a linear model: home range size~mean distance between encounters with prey herds. Data for some of the lions were collected for more than one season, and in such cases, we used the mean of the repeated measures in our analysis. For the subsequent analyses, we used generalized linear models with a quasi‐Poisson distribution to analyze pride size and subgroup size. To test the third prediction, we fitted two models: pride size ~ coefficient of variation in the distance between prey herds and mean subgroup size ~ coefficient of variation in the distance between prey herds. To test the fourth prediction, we fitted four models: pride size ~ mean prey herd size, pride size ~ mean prey body size, mean subgroup size ~ mean prey herd size, and mean subgroup size ~ mean prey body size. Finally, to test our fifth hypothesis, we fitted a model: frequency of pride fission ~ pride size. This allowed us to get an understanding of whether prides fission more when in larger prides (VanderWaal, Mosser, & Packer, 2009).

## 3 RESULTS

The sizes of lion prides (number of adult males and females) ranged between 1 and 11 lions, with a mean ± *SD* = 4.8 ± 2.5 lions. The subgroup sizes ranged between 1 and 11 lions, with a mean ± *SD* = 2.4 ± 1.6 lions. The home range sizes ranged between 23 km^2^ and 1,511 km^2^, with a mean ± *SD* = 344 km^2^ ± 403 km^2^.

### 3.1 Home range size is independent of group size

**Figure 2:**
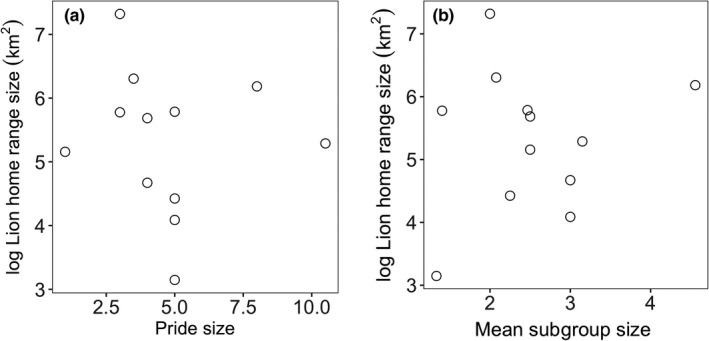
Female lion seasonal home range size had no significant relationship with (a) pride size and with (b) mean subgroup size

The relationship between pride size and home range size was not significant (*F*_1,10_ = .10; *p* = .76; *R* ^2^ = −.09; Figure 2a). The relationship between mean subgroup size and home range size was also not significant (*F*_1,10_ = .24; *p* = .63; *R* ^2^ = −.07; Figure 2b).

### 3.2 Home range size increases with resource dispersion

**Figure 3:**
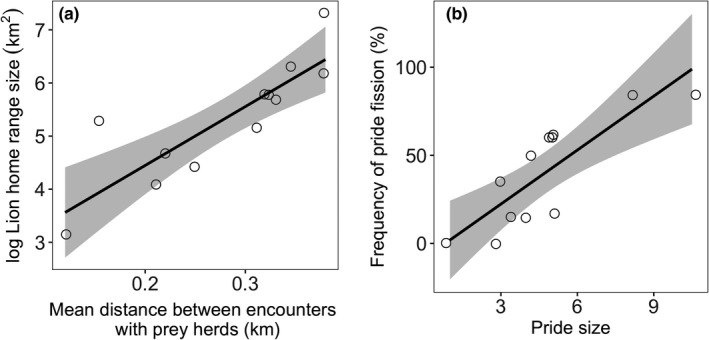
(a) Female lion seasonal home range size increased with increase in the index of prey dispersion (mean distance between encounters with prey herds). (b) Frequency of pride fission (the number of times the pride was in subgroups as a proportion of the total number of whole pride and subgroup sightings) increased with increase in pride size

Home range size increased with increase in the mean distance between encounters with prey herds, an index of prey dispersion (*F*_1,10_ = 26.96; *p* < .001; *R* ^2^ = .70; Figure 3a).

### 3.3 Group size is not significantly influenced by resource heterogeneity

The relationship between the index of resource heterogeneity, coefficient of variation in the distance between prey herds, and pride size was not significant (estimate ± *SE* = −.091 ± .165; *t*(10) = −0.09; *p* = .60). Again, the relationship between the index of resource heterogeneity, coefficient of variation in the distance between prey herds, and mean subgroup size was not significant (estimate ± *SE* = −.041 ± .105; *t*(10) = −0.40; *p* = .70).

### 3.4 Group size is not significantly influenced by richness of resource patches

None of the indices of patch richness—mean prey body size (estimate ± *SE* = .002 ± .002; *t*(10) = 0.88; *p* = .40) and mean prey herd size (estimate ± *SE* = .061 ± .053; *t*(10) = 1.17; *p* = .27)—had a significant relationship with pride size. Again, none of the indices of patch richness—mean prey body size (estimate ± *SE* = −.0005 ± .001; *t*(10) = −0.33; *p* = .75) and mean prey herd size (estimate ± *SE* = .051 ± .031; *t*(10) = 1.66; *p* = .13)—had a significant relationship with mean subgroup size.

### 3.5 Fission–fusion dynamics is influenced by group size

The frequency of pride fission significantly increased with increase in pride size (estimate ± *SE* = .203 ± .059; *t*(10) = 3.43; *p* = .006; Figure 3b).

## 4 DISCUSSION

Our study offers new insights into the relevance of the resource dispersion hypothesis to a fission–fusion society. The first two predictions of the RDH gained straightforward support from our study of lions. First, neither pride size nor mean subgroup size had an effect on home range size (first prediction). In a null hypothesis, as might be expected in an homogeneous landscape, territory would expand to meet the increased metabolic requirements of larger groups (Johnson, Macdonald, Newman, & Morecroft, 2001; see Kruuk & Macdonald, 1985 for a discussion of this mechanism, known as “expansionism” within the RDH). However, we found no such correlation between lion home range size and group size, similarly to other previous studies (see Johnson et al., 2002 for a review), which suggest that these two variables are linked to different, likely independent, factors. In line with the second prediction, home range size increased with prey dispersion as measured by how herds of prey were spread out through the landscape. The dispersion of prey herds emerged as an important factor in shaping lion home range size. For a given patch richness, one would expect territory size to be larger where patches are more dispersed, that is, fewer patches per unit area (Johnson et al., 2002). Lions are expected to expand their home ranges to encompass enough prey herds (resource patches), so that at one time, at least one prey herd has an adequate level of food security, and lions can rotate their hunting between prey herds within home ranges (Carr & Macdonald, 1986; Valeix et al., 2012).

The unpredictability in the availability of resource patches is expected to influence group size, with group size increasing with increased variability in the availability of resource aggregations (Johnson et al., 2002). However, this third prediction of the RDH was not supported by our study where the heterogeneity in the distances between prey herds did not influence lion group size. This could be because the overall abundance in resource patches should be the same for this prediction to hold (Johnson et al., 2002), or because this particular measure of heterogeneity in resource availability is not the one that most impacts lions. Besides, this study, together with some previous RDH studies, failed to find a clear relationship between patch richness and group size, and hence straightforward support for the fourth RDH prediction. Despite the intensity of fieldwork, scale of study area, and the relatively large data set, our descriptors of pride dynamics (and indeed of resource patch dynamics) remain coarse reflections of the naturalistic reality. Both the size of the prey herd and the size of the prey individuals comprising it might plausibly be thought of as measures of patch richness insofar as it is obvious in lion natural history that both will have potential impact on the number of lions that can feed together. However, prey size as a measure of patch richness is also complicated in that the availability of large prey does not always translate to profitability, as some large prey may be too risky to hunt safely (Elliott, Cowan, & Holling, 1977; Makacha & Schaller, 1969). This challenge in measuring patch resource richness is one of the reasons why the RDH has not been appropriately tested or applied (Johnson et al., 2002). Additionally, group size has fitness benefits apart from resources, and grouping patterns of lions can result from other factors such as the demands of protecting their young and themselves against encounters with neighboring prides (Mosser & Packer, 2009; Packer, 1986) and maintaining a long‐term territory (Mosser & Packer, 2009; Packer, Scheel, & Pusey, 1990).

We confirm, albeit with advanced satellite technology and large sample sizes, Schaller's (1972) observation that larger prides have a higher frequency of fission than smaller prides. The predatory strategy of lions has been classified as group foraging with inconsistent membership, and resources shared by all individuals in a group (Lang & Farine, 2017). Nonetheless, high spatial variability in resource availability would increase the chances of conflict between group members (Sueur et al., 2011) as they compete for access to the unpredictable resources (Krause & Ruxton, 2002). This can favor the complete or partial separation of individuals into subgroups. We conclude that fission–fusion in larger prides is an adaptation to the spatial and temporal variability in food resources (Couzin & Laidre, 2009; Lehmann, Korstjens, & Dunbar, 2007). Remembering Kruuk's (1972) insight that the size of hunting parties of spotted hyenas was decided by the species of prey they intended to hunt, it is plausible that the size of subgroup hunting parties into which a large pride fissions is determined by the body size of the available prey that being one of the two dimensions we explore of the richness of patches.

As an interpretation to be tested in further studies, we suggest that the relationship between pride size and prey size can have two aspects (cause and effect) and which of these predominates is likely to change with its size during a pride's history. Lions seek to grow their prides for collective strength, as larger prides are significantly more likely to maintain control of disputed areas and to improve the quality of their territories (Mosser & Packer, 2009). As the pride grows, it becomes large enough that all members can more readily cooperate to kill very large prey (Schaller, 1972), and this offers the best per capita return on investment for each member (a metric pioneered by Caraco & Wolf, 1975; see also Stander, 1992). However, as the pride grows beyond a limit where patch richness sets the upper limit to its size (Valeix et al., 2012), each prey item provides an inadequate meal for the whole pride so they tend to fission into smaller hunting parties (see Van Orsdol, 1984), and perhaps paradoxically, this leads to a larger total pride size comprised of a greater number of smaller hunting parties. It may be that these hunting parties are most efficient when hunting medium‐sized prey, although doubtless occasionally killing very large prey when in larger hunting parties (Packer et al., 1990). At this point in the growth of an hypothetical pride from small to large, pride size is not determined by patch richness, rather hunting party size has an effect on the relationship between prey size and each member of the hunting party's return on investment (Stander, 1992). One can interpret the fission–fusion dynamics as an adaptive mechanism for reducing competition within the group (Couzin & Laidre, 2009; Lehmann & Boesch, 2004), allowing larger prides to be supported even when prey sizes are medium. Equally, from a starting point at which other socio‐ecological factors have favored the development of very large prides, fission–fusion enables hunting parties of a size that maximizes individual return on investment. Clearly, the next priority in the exploration of the RDH is to measure the size of hunting parties from observation of hunts and to relate those to diverse measures of resource dispersion. Additionally, how the fission–fusion dynamics affect collective decision making in social animals is a rich vein for further study (Sueur et al., 2011).

Transposing RDH predictions into measures capturing realistically the complexity of animals living in the wild is challenging. Acknowledging that complexity, our study has limitations. Although we attempted to account for factors beyond resource dispersion that surely affect grouping behaviors in lions (e.g., reproductive status), there are obvious confounds that are so far not addressed (e.g., the presence and interactions with neighboring prides). Although temporal heterogeneity in resource availability may also have an influence on group size, we could not measure this because of the small sample size. Further studies will need to be carried out to determine the effect of fission–fusion dynamics on the relationship between group size and patch richness. Failure to include these dynamics may explain why the relationship between group size and patch richness has not been clear in some previous RDH studies. Whatever larger theoretical framework may emerge to explain lion society, we believe that by incorporating fission–fusion dynamics the RDH can be refined and improved.

## CONFLICT OF INTEREST

None declared.

## AUTHORS' CONTRIBUTIONS

M.M.M., M.V., D.W.M., and A.J.L. conceived the ideas and designed the methodology; M.M.M. collected the data; M.M.M and M.V analyzed the data; and M.M.M. led the writing of the manuscript. All authors contributed critically to the drafts and gave final approval for publication.

## Data Availability

The data supporting the results are achieved on Dryad (https://doi.org/10.5061/dryad.4h6h6p5).

## References

[ece35456-bib-0001] Calenge, C. (2006). The package “adehabitat” for the R software: A tool for the analysis of space and habitat use by animals. Ecological Modelling, 197, 516–519. 10.1016/j.ecolmodel.2006.03.017

[ece35456-bib-0002] Caraco, T. , & Wolf, L. L. (1975). Ecological determinants of group sizes of foraging lions. The American Naturalist, 109, 343–352. 10.1086/283001

[ece35456-bib-0003] Carr, G. M. , & Macdonald, D. W. (1986). The sociality of solitary foragers: A model based on resource dispersion. Animal Behaviour, 34, 1540–1549. 10.1016/S0003-3472(86)80223-8

[ece35456-bib-0004] Chamaillé‐Jammes, S. , Charbonnel, A. , Dray, S. , Madzikanda, H. , & Fritz, H. (2016). Spatial distribution of a large herbivore community at waterholes: an assessment of its stability over years in Hwange National Park, Zimbabwe. PLoS ONE, 11(4), e0153639. 27074044 10.1371/journal.pone.0153639 PMC4830562

[ece35456-bib-0005] Conradt, L. , & Roper, T. J. (2005). Consensus decision making in animals. Trends in Ecology & Evolution, 20, 449–456. 10.1016/j.tree.2005.05.008 16701416

[ece35456-bib-0006] Couzin, I. D. , & Laidre, M. E. (2009). Fission‐fusion populations. Current Biology, 19, R633–R635. 10.1016/j.cub.2009.05.034 19674541

[ece35456-bib-0007] Cumming, D. H. M. , & Cumming, G. S. (2003). Ungulate community structure and ecological processes: Body size, hoof area and trampling in African savannas. Oecologia, 134, 560–568. 10.1007/s00442-002-1149-4 12647129

[ece35456-bib-0008] Elliott, J. P. , Cowan, I. M. , & Holling, C. S. (1977). Prey capture by the African lion. Canadian Journal of Zoology, 55, 1811–1828. 10.1139/z77-235

[ece35456-bib-0009] Funston, P. J. , Frank, L. , Stephens, T. , Davidson, Z. , Loveridge, A. , Macdonald, D. M. , … Ferreira, S. M. (2010). Substrate and species constraints on the use of track incidences to estimate African large carnivore abundance. Journal of Zoology, 281, 56–65. 10.1111/j.1469-7998.2009.00682.x

[ece35456-bib-0010] Groenendijk, J. , Hajek, F. , Schenck, C. , Staib, E. , Johnson, P. J. , & Macdonald, D. W. (2015). Effects of territory size on the reproductive success and social system of the giant otter, south‐eastern Peru. Journal of Zoology, 296, 153–160. 10.1111/jzo.12231

[ece35456-bib-0011] Hemson, G. , Johnson, P. , South, A. , Kenward, R. , Ripley, R. , & Macdonald, D. (2005). Are kernels the mustard? Data from global positioning system (GPS) collars suggests problems for kernel home‐range analyses with least‐squares cross‐validation. Journal of Animal Ecology, 74, 455–463. 10.1111/j.1365-2656.2005.00944.x

[ece35456-bib-0012] Holmes, S. M. , Gordon, A. D. , Louis, E. E. , & Johnson, S. E. (2016). Fission‐fusion dynamics in black‐and‐white ruffed lemurs may facilitate both feeding strategies and communal care of infants in a spatially and temporally variable environment. Behavioral Ecology and Sociobiology, 70, 1949–1960. 10.1007/s00265-016-2201-4

[ece35456-bib-0013] Johnson, D. D. P. , Kays, R. , Blackwell, P. G. , & Macdonald, D. W. (2002). Does the resource dispersion hypothesis explain group living? Trends in Ecology & Evolution, 17, 563–570. 10.1016/S0169-5347(02)02619-8

[ece35456-bib-0014] Johnson, D. D. P. , Macdonald, D. W. , Newman, C. , & Morecroft, M. D. (2001). Group size versus territory size in group‐living badgers: A large‐sample field test of the resource dispersion hypothesis. Oikos, 95, 265–274. 10.1034/j.1600-0706.2001.950208.x PMC37404 11511326

[ece35456-bib-0015] Kerth, G. , Ebert, C. , & Schmidtke, C. (2006). Group decision making in fission‐fusion societies: Evidence from two‐field experiments in Bechstein's bats. Proceedings of the Royal Society B: Biological Sciences, 273, 2785–2790. 10.1098/rspb.2006.3647 PMC1635504 17015328

[ece35456-bib-0016] Krause, J. , & Ruxton, G. D. (2002). Living in groups. Oxford, UK: Oxford University Press.

[ece35456-bib-0017] Kruuk, H. (1972). The spotted hyaena. A Study of predation and social behaviour. Chicago, IL: University of Chicago Press.

[ece35456-bib-0018] Kruuk, H. (1978). Foraging and spatial‐organization of European badger, *Meles‐meles L* . Behavioral Ecology and Sociobiology, 4, 75–89.

[ece35456-bib-0019] Kruuk, H. , & Macdonald, D. W. (1985). Group territories of carnivores: Empires and enclaves In Sibly R. M., & Smith R. H. (Eds.), Behavioural ecology (pp. 521–536). Oxford, UK: Blackwell Scientific Publications.

[ece35456-bib-0020] Lang, S. D. J. , & Farine, D. R. (2017). A multidimensional framework for studying social predation strategies. Nature Ecology & Evolution, 1, 1230–1239. 10.1038/s41559-017-0245-0 29046557

[ece35456-bib-0021] Lehmann, J. , & Boesch, C. (2004). To fission or to fusion: Effects of community size on wild chimpanzee (Pan troglodytes verus) social organisation. Behavioral Ecology and Sociobiology, 56, 207–216. 10.1007/s00265-004-0781-x

[ece35456-bib-0022] Lehmann, J. , Korstjens, A. H. , & Dunbar, R. I. M. (2007). Fission–fusion social systems as a strategy for coping with ecological constraints: A primate case. Evolutionary Ecology, 21, 613–634. 10.1007/s10682-006-9141-9

[ece35456-bib-0023] Loveridge, A. J. , Hunt, J. E. , Murindagomo, F. , & Macdonald, D. W. (2006). Influence of drought on predation of elephant (*Loxodonta africana*) calves by lions (*Panthera leo*) in an African wooded savannah. Journal of Zoology, 270, 523–530. 10.1111/j.1469-7998.2006.00181.x

[ece35456-bib-0024] Macdonald, D. W. (1981). Resource dispersion and the social organization of the red fox (*Vulpes vulpes*) In Chapman J. A., & Pursely D. (Eds.), Proceedings of the Worldwide Furbearer Conference (pp. 918–949). College Park, MD: University of Maryland Press.

[ece35456-bib-0025] Macdonald, D. W. (1983). The ecology of carnivore social‐behavior. Nature, 301, 379–384.

[ece35456-bib-0026] Macdonald, D. W. , & Johnson, D. D. P. (2015). Patchwork planet: The resource dispersion hypothesis, society, and the ecology of life. Journal of Zoology, 295, 75–107. 10.1111/jzo.12202

[ece35456-bib-0027] Makacha, S. , & Schaller, G. B. (1969). Observations on lions in the Lake Manyara National Park, Tanzania. African Journal of Ecology, 7, 99–103. 10.1111/j.1365-2028.1969.tb01198.x

[ece35456-bib-0028] Mills, M. G. L. (1982). Factors affecting group‐size and territory size of the brown hyena, *hyaena‐brunnea* in the Southern Kalahari. Journal of Zoology, 198, 39–51.

[ece35456-bib-0029] Mosser, A. , Fryxell, J. M. , Eberly, L. , & Packer, C. (2009). Serengeti real estate: Density vs. fitness‐based indicators of lion habitat quality. Ecology Letters, 12, 1050–1060. 10.1111/j.1461-0248.2009.01359.x 19708970

[ece35456-bib-0030] Mosser, A. , & Packer, C. (2009). Group territoriality and the benefits of sociality in the African lion, *Panthera leo* . Animal Behaviour, 78, 359–370. 10.1016/j.anbehav.2009.04.024

[ece35456-bib-0031] Packer, C. (1986). The ecology of sociality in felids In Rubenstein D. I., & Wrangham R. W. (Eds.), Ecological aspects of social evolution: Birds and mammals (pp. 429–452). Princeton, NJ: Princeton University Press.

[ece35456-bib-0032] Packer, C. , & Pusey, A. E. (1982). Cooperation and competition within coalitions of male lions: Kin selection or game‐theory? Nature, 296, 740–742. 10.1038/296740a0

[ece35456-bib-0033] Packer, C. , & Pusey, A. E. (1997). Divided we fall: Cooperation among lions. Scientific American, 276, 52–59. 10.1038/scientificamerican0597-52 8972618

[ece35456-bib-0034] Packer, C. , Scheel, D. , & Pusey, A. E. (1990). Why lions form groups – Food is not enough. The American Naturalist, 136, 1–19. 10.1086/285079

[ece35456-bib-0035] Powell, R. A. (2000). Animal home ranges and territories and home range estimators In Boitani L., & Fuller T. K. (Eds.), Research techniques in animal ecology controversies and consequences (pp. 65–110). Columbia, SC: Columbia University Press.

[ece35456-bib-0036] QGIS Development Team (2019). QGIS Geographic Information System. Open Source. Geospatial Foundation Project.

[ece35456-bib-0037] R Core Team (2019). R: A language and environment for statistical computing. Vienna, Austria: R Foundation for Statistical Computing.

[ece35456-bib-0038] Redfern, J. V. , Grant, R. , Biggs, H. , & Getz, W. M. (2003). Surface‐water constraints on herbivore foraging in the Kruger National Park, South Africa. Ecology, 84, 2092–2107. 10.1890/01-0625

[ece35456-bib-0039] Schaller, G. B. (1972). The Serengeti lion; a study of predator‐prey relations. Chicago, IL: University of Chicago Press.

[ece35456-bib-0040] Silk, M. J. , Croft, D. P. , Tregenza, T. , & Bearhop, S. (2014). The importance of fission–fusion social group dynamics in birds. Ibis, 156, 701–715. 10.1111/ibi.12191

[ece35456-bib-0041] Silveira, L. , Jacomo, A. T. A. , & Diniz, J. A. F. (2003). Camera trap, line transect census and track surveys: A comparative evaluation. Biological Conservation, 114, 351–355. 10.1016/S0006-3207(03)00063-6

[ece35456-bib-0042] Stander, P. E. (1992). Foraging dynamics of lions in a semi‐arid environment. Canadian Journal of Zoology, 70, 8–21. 10.1139/z92-002

[ece35456-bib-0043] Sueur, C. , King, A. J. , Conradt, L. , Kerth, G. , Lusseau, D. , Mettke‐Hofmann, C. , … Aureli, F. (2011). Collective decision‐making and fission–fusion dynamics: A conceptual framework. Oikos, 120, 1608–1617. 10.1111/j.1600-0706.2011.19685.x

[ece35456-bib-0044] Symington, M. M. (1990). Fission‐fusion social organization in Ateles and Pan. International Journal of Primatology, 11, 47–61. 10.1007/BF02193695

[ece35456-bib-0045] Valeix, M. , Loveridge, A. J. , Chamaille‐Jammes, S. , Davidson, Z. , Murindagomo, F. , Fritz, H. , & Macdonald, D. W. (2009). Behavioral adjustments of African herbivores to predation risk by lions: Spatiotemporal variations influence habitat use. Ecology, 90, 23–30. 10.1890/08-0606.1 19294909

[ece35456-bib-0046] Valeix, M. , Loveridge, A. J. , Davidson, Z. , Madzikanda, H. , Fritz, H. , & Macdonald, D. W. (2010). How key habitat features influence large terrestrial carnivore movements: Waterholes and African lions in a semi‐arid savanna of north‐western Zimbabwe. Landscape Ecology, 25, 337–351. 10.1007/s10980-009-9425-x

[ece35456-bib-0047] Valeix, M. , Loveridge, A. J. , & Macdonald, D. W. (2012). Influence of prey dispersion on territory and group size of African lions: A test of the resource dispersion hypothesis. Ecology, 93, 2490–2496. 10.1890/12-0018.1 23236920

[ece35456-bib-0048] Van Orsdol, K. G. (1984). Foraging behavior and hunting success of lions in Queen Elizabeth National Park, Uganda. African Journal of Ecology, 22, 79–99.

[ece35456-bib-0049] VanderWaal, K. L. , Mosser, A. , & Packer, C. (2009). Optimal group size, dispersal decisions and postdispersal relationships in female African lions. Animal Behaviour, 77, 949–954. 10.1016/j.anbehav.2008.12.028

[ece35456-bib-0050] Venables, W. N. , & Ripley, B. D. (2002). Modern applied statistics with S, 4th ed. New York, NY: Springer.

[ece35456-bib-0051] Wilson, G. J. , & Delahay, R. J. (2001). A review of methods to estimate the abundance of terrestrial carnivores using field signs and observation. Wildlife Research, 28, 151–164. 10.1071/WR00033

